# Adverse effects of hypertension, supine hypertension, and perivascular space on cognition and motor function in PD

**DOI:** 10.1038/s41531-021-00214-6

**Published:** 2021-08-10

**Authors:** Na-Young Shin, Yae Won Park, Sang-Won Yoo, Ji-Yeon Yoo, Yangsean Choi, Jinhee Jang, Kook-Jin Ahn, Bum-soo Kim, Joong-Seok Kim

**Affiliations:** 1 Department of Radiology, Seoul St. Mary’s Hospital, College of Medicine, The Catholic University of Korea, Seoul, Korea; 2 Department of Radiology and Research Institute of Radiological Science and Center for Clinical Imaging Data Science, Yonsei University College of Medicine, Seoul, Korea; 3 Department of Neurology, Seoul St. Mary’s Hospital, College of Medicine, The Catholic University of Korea, Seoul, Korea

**Keywords:** Diagnostic markers, Parkinson's disease

## Abstract

Dilated perivascular space (dPVS) has recently been reported as a biomarker for cognitive impairment in Parkinson’s disease (PD). However, comprehensive interrelationships between various clinical risk factors, dPVS, white-matter hyperintensities (WMH), cognition, and motor function in PD have not been studied yet. The purpose of this study was to test whether dPVS might mediate the effect of clinical risk factors on WMH, cognition, and motor symptoms in PD patients. A total of 154 PD patients were assessed for vascular risk factors (hypertension, diabetes mellitus, and dyslipidemia), autonomic dysfunction (orthostatic hypotension and supine hypertension [SH]), APOE *ε*4 genotype, rapid eye movement sleep-behavior disorder, motor symptoms, and cognition status. The degree of dPVS was evaluated in the basal ganglia (BG) and white matter using a 5-point visual scale. Periventricular, deep, and total WMH severity was also assessed. Path analysis was performed to evaluate the associations of these clinical factors and imaging markers with cognitive status and motor symptoms. Hypertension and SH were significantly associated with more severe BGdPVS, which was further associated with higher total WMH, consequently leading to lower cognitive status. More severe BGdPVS was also associated with worse motor symptoms, but without mediation of total WMH. Similar associations were seen when using periventricular WMH as a variable, but not when using deep WMH as a variable. In conclusion, BGdPVS mediates the effect of hypertension and SH on cognitive impairment via total and periventricular WMH, while being directly associated with more severe motor symptoms.

## Introduction

Parkinson’s disease (PD) has been primarily considered a motor disorder, but cognitive impairment is also a common clinical manifestation^1^. Various clinical and imaging biomarkers have been reported as risk factors for cognitive impairment and motor symptoms in PD. Vascular risk factors and imaging markers representing small-vessel disease burden, such as white-matter hyperintensities (WMH), have been seen to adversely affect cognition and motor functions in PD^2,3^. Additionally, several nonmotor symptoms, such as olfactory dysfunction^4,5^, depression^6^, autonomic dysfunction such as orthostatic hypotension (OH) and supine hypertension (SH)^1,4,7^, and rapid eye movement sleep-behavior disorder (RBD)^5^, have also been suggested as risk factors for cognitive and motor impairment in PD. APOE *ε*4 allele has also shown association with cognitive impairment in PD^8^.

Perivascular spaces are fluid-filled cavities surrounding the small penetrating cerebral arterioles and venules. When visible on MRI, these are considered to be dilated and functionally impaired. Recently, dilated perivascular spaces (dPVS), particularly those located in the basal ganglia (BGdPVS), have been emerging as an imaging marker for cerebral small-vessel disease^9^. Increasing evidence suggests possible links between dPVS, WMH, clinical risk factors, and cognitive and motor symptoms in PD. In a longitudinal study^10^, the progression of WMH was associated with higher BGdPVS burden at baseline. Among clinical risk factors, hypertension is a well-established risk factor of BGdPVS as well as of WMH^9^. OH^11,12^, and SH^11^, occasionally accompanied with OH, have also been associated with WMH. The association between sleep quality and dPVS^13^ suggests the possibility of RBD as a risk factor for dPVS. The APOE *ε*4 allele is a risk factor for WMH^14^ and has also been associated with dPVS^15^. Moreover, a recent longitudinal study reported BGdPVS to be a predictor for cognitive impairment in PD^16^, and a few case reports have suggested an association between dPVS and parkinsonism^17,18^. Thus, we can postulate that dPVS plays a potential role in mediating the effect of clinical risk factors on WMH and cognitive and motor symptoms in PD.

However, to the best of our knowledge, no study has evaluated on the comprehensive interrelationships between clinical risk factors, dPVS, WMH, cognition, and motor function in PD. We therefore performed path analyses to evaluate whether dPVS mediates the effects of clinical risk factors on cognition and motor function in PD.

## Results

### Demographic characteristics

Among the 154 patients, who were predominantly drug-naive PD (*n* = 116), 36 were diagnosed as PD-IC, 111 as PD-MCI, and seven as PDD. PDD patients had more severe motor symptoms than PD-IC patients. General cognitive function was the highest in the PD-IC patients, followed by the PD-MCI and PDD patients. Among the imaging markers, PDD patients showed more severe BGdPVS than PD-IC and PD-MCI patients. PDD patients had higher periventricular WMH burden than PD-IC patients (Table 1). Detailed information on dPVS and WMH scores according to cognitive status is shown in Supplementary Table 1.

**Table 1:** Clinical and imaging characteristics of patients.

	PD-IC (*n* = 36)	PD-MCI (*n* = 111)	PDD (*n* = 7)	*P*	*P*^a *^	*P*^b *^	*P*^c *^
*Clinical variables*							
Age, y	68.7 ± 7.9	70.2 ± 9.3	76.9 ± 7.2	0.085			
Age at onset, y	65 (59–69)	67 (60–73)	73 (68–78)	0.092			
Male	16 (44.4)	59 (53.2)	4 (57.1)	0.630			
Education, y	12 (11–16)	12 (6–15)	12 (9–17)	0.189			
Parkinsonism duration, m	12 (7–28)	12 (6–24)	24 (8–30)	0.777			
Levodopa-equivalent dose, mg	0 (0–0)	0 (0–225)	0 (0–150)	0.097			
UPDRS III	13 (13–20)	15 (10–21)	24 (19–27)	0.041	0.999	0.037	0.069
MMSE	29 (28–30)	27 (26–29)	23 (22–25)	<0.001	0.001	<0.001	0.005
CCSIT	6.8 ± 2.4	6.0 ± 2.7	5.7 ± 3.0	0.250			
Depression	21 (58.3)	57 (51.4)	6 (85.7)	0.182			
OH	6 (16.7)	22 (19.8)	2 (28.6)	0.756			
SH	1 (2.8)	11 (9.9)	1 (14.3)	0.348			
RBD	8 (22.2)	44 (39.6)	2 (28.6)	0.153			
APOE ε4 carrier	7 (19.4)	22 (18.8)	1 (14.3)	0.938			
*Vascular risk factors*							
Hypertension	17 (47.2)	45 (40.6)	4 (57.1)	0.575			
Diabetes mellitus	9 (25.0)	15 (13.5)	3 (42.9)	0.062			
Dyslipidemia	22 (61.1)	56 (50.5)	3 (42.9)	0.468			
*Imaging findings*							
BGdPVS	1 (1–2)	2 (1–2)	3 (2–4)	0.010	0.901	0.009	0.027
WMdPVS	3 (2–4)	3 (2–4)	3 (2–4)	0.779			
Total WMH	2 (1–2)	2 (2–3)	4 (2–4)	0.016	0.055	0.102	0.493
Periventricular WMH	1 (1–1)	1 (1–2)	3 (1–3)	0.020	0.160	0.047	0.215
Deep WMH	1 (0–1)	1 (1–1)	1 (1–1)	0.106			

Data are expressed as means ± standard deviations or medians with interquartile ranges or as numbers with percentages in parentheses.

*P* refers to the statistical significance among the three groups.

^*^P^*a*^*, P*^*b*^, and *P*^*c*^ refer to the corrected statistical significance with Bonferroni-like adjustment for differences between the PD-IC and PD-MCI, PD-IC and PDD, and PD-MCI and PDD groups, respectively.

Abbreviations: BG basal ganglia, CCSIT Cross-Cultural Smell Identification Test, dPVS dilated perivascular space, MMSE Mini-Mental State Examination, OH orthostatic hypotension, PDD PD with dementia, PD-IC PD with intact cognition, PD-MCI PD with mild cognitive impairment, RBD REM sleep behavior disorder, SH supine hypertension, UPDRS III Unified Parkinson’s Disease Rating Scale III, WM white matter, WMH white matter hyperintensities.

Interobserver reliability was good both for BGdPVS (*κ* = 0.74) and white matter dPVS (WMdPVS) (*κ* = 0.71).

### Path analyses

The variable-inflation factors were below four for all variables, so we assumed that our variables had a low degree of multicollinearity.

#### Prediction of cognitive status

**Fig. 1:**
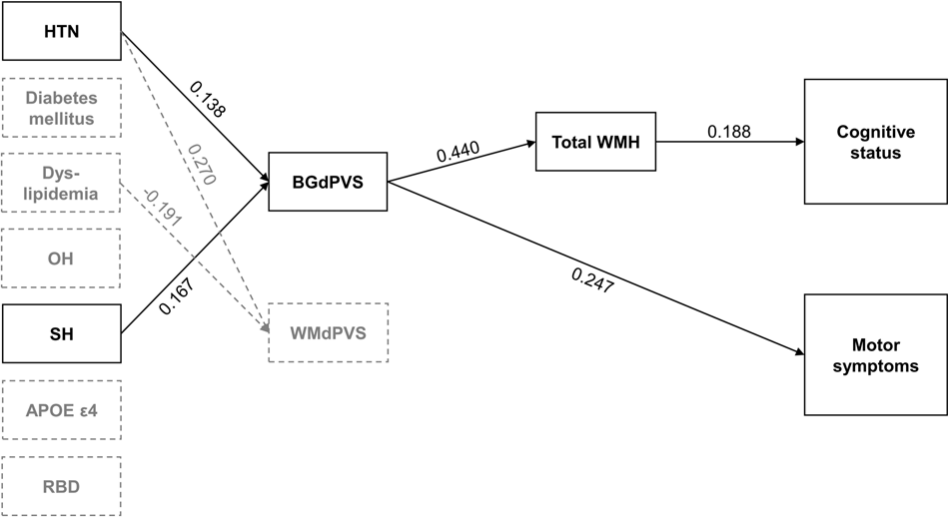
Schematic diagram of path analyses for cognitive status and motor symptoms. Vascular risk factors (hypertension, diabetes mellitus, and dyslipidemia), autonomic risk factors (OH, SH), RBD, and APOE ε4 status were entered as predictors. BGdPVS, WMdPVS, and total WMH were entered as mediators. Age, sex, years of education, disease duration, levodopa-equivalent dosage, CCSIT score, and the presence of depression were entered as covariates. Numbers on the paths are the standardized coefficients that were statistically significant. Abbreviations: BG basal ganglia, dPVS dilated perivascular spaces, CCSIT Cross-Cultural Smell Identification Test, HTN hypertension, OH orthostatic hypotension, RBD REM sleep-behavior disorder, SH supine hypertension, WM white-matter, WMH white matter hyperintensities.

When total WMH was used as a variable, hypertension (*β* = 0.138, *P* = 0.045) and SH (*β* = 0.167, *P* = 0.016) were significantly associated with severe BGdPVS. Severe BGdPVS (*β* = 0.440, *P* < 0.001) was further associated with higher total WMH, and higher total WMH (*β* = 0.188, *P* = 0.047) was consequently related to poor cognitive status. Hypertension (*β* = 0.270, *P* < 0.001) and absence of dyslipidemia (*β* =− 0.191, *P* = 0.013) were associated with severe WMdPVS, but WMdPVS was not significantly associated with WMH nor cognitive status (Table 2, Fig. 1).

**Table 2:** Effects of predictors on cognitive status and motor symptoms through mediators when total WMH was used as a variable.

	BGdPVS			WMdPVS			Total WMH			Cognitive status			UPDRSIII		
	*β*	SE	*P*	*β*	SE	*P*	*β*	SE	*P*	*β*	SE	*P*	*β*	SE	*P*
Hypertension	0.138	0.148	0.045	0.270	0.158	<0.001	0.033	0.153	0.640	−0.026	0.083	0.754	−0.126	1.435	0.097
Diabetes mellitus	−0.071	0.194	0.313	−0.140	0.206	0.069	0.029	0.193	0.674	−0.030	0.104	0.711	0.014	1.808	0.852
Dyslipidemia	−0.070	0.148	0.318	−0.191	0.157	0.013	0.013	0.148	0.847	−0.066	0.080	0.415	−0.100	1.391	0.179
OH	−0.097	0.190	0.170	−0.025	0.202	0.744	−0.073	0.187	0.289	0.020	0.102	0.806	0.120	1.762	0.109
SH	0.167	0.266	0.016	0.083	0.283	0.276	0.036	0.266	0.599	0.069	0.145	0.396	−0.047	2.501	0.529
RBD	0.047	0.160	0.514	0.079	0.170	0.316	−0.003	0.157	0.971	0.111	0.085	0.173	0.032	1.474	0.675
APOE ε4 carrier	0.103	0.183	0.136	−0.052	0.195	0.491	0.00	0.181	0.916	−0.023	0.098	0.776	−0.088	1.697	0.225
BGdPVS							0.440	0.079	<0.001	0.044	0.047	0.664	0.247	0.810	0.007
WMdPVS							−0.031	0.074	0.663	−0.020	0.040	0.812	−0.105	0.695	0.168
Total WMH										0.188	0.044	0.047	−0.046	0.753	0.593

Age, sex, years of education, disease duration, levodopa-equivalent dose, CCSIT score, and the presence of depression were used as covariates.

Abbreviations: *β* standardized beta coefficient, BG basal ganglia, dPVS dilated perivascular spaces, OH orthostatic hypotension, RBD REM sleep behavior disorder, SE standard error, SH supine hypertension, UPDRS III Unified Parkinson’s Disease Rating Scale III, WM white matter, WMH white matter hyperintensities.

Similar results were found when periventricular WMH was used as a variable instead of total WMH, showing significant associations between severe BGdPVS (*β* = 0.443, *P* < 0.001) and higher periventricular WMH, and between higher periventricular WMH (*β* = 0.199, *P* = 0.048) and poor cognitive status (Supplementary Table 2, Supplementary Figure 1).

When deep WMH was used as a variable, severe BGdPVS (*β* = 0.293, *P* = 0.001) and absence of OH (*β* =− 0.218, *P* = 0.005) were associated with higher deep WMH, but there was no significant association between deep WMH and cognitive status (Supplementary Table 3).

#### Prediction of motor symptoms

When total WMH was used as a variable, severe BGdPVS (*β* = 0.247, *P* = 0.007) was associated with severe motor symptoms without being mediated by total WMH (Table 2, Fig. 1).

When either periventricular WMH or deep WMH was used as a variable instead of total WMH, severe BGdPVS (*β* = 0.210, *P* = 0.025 or *β* = 0.255, *P* = 0.003, respectively) was associated with more severe motor symptoms without being mediated by periventricular WMH or deep WMH, respectively (Supplementary Tables 2 and 3, Supplementary Fig. 1).

**Fig. 2:**
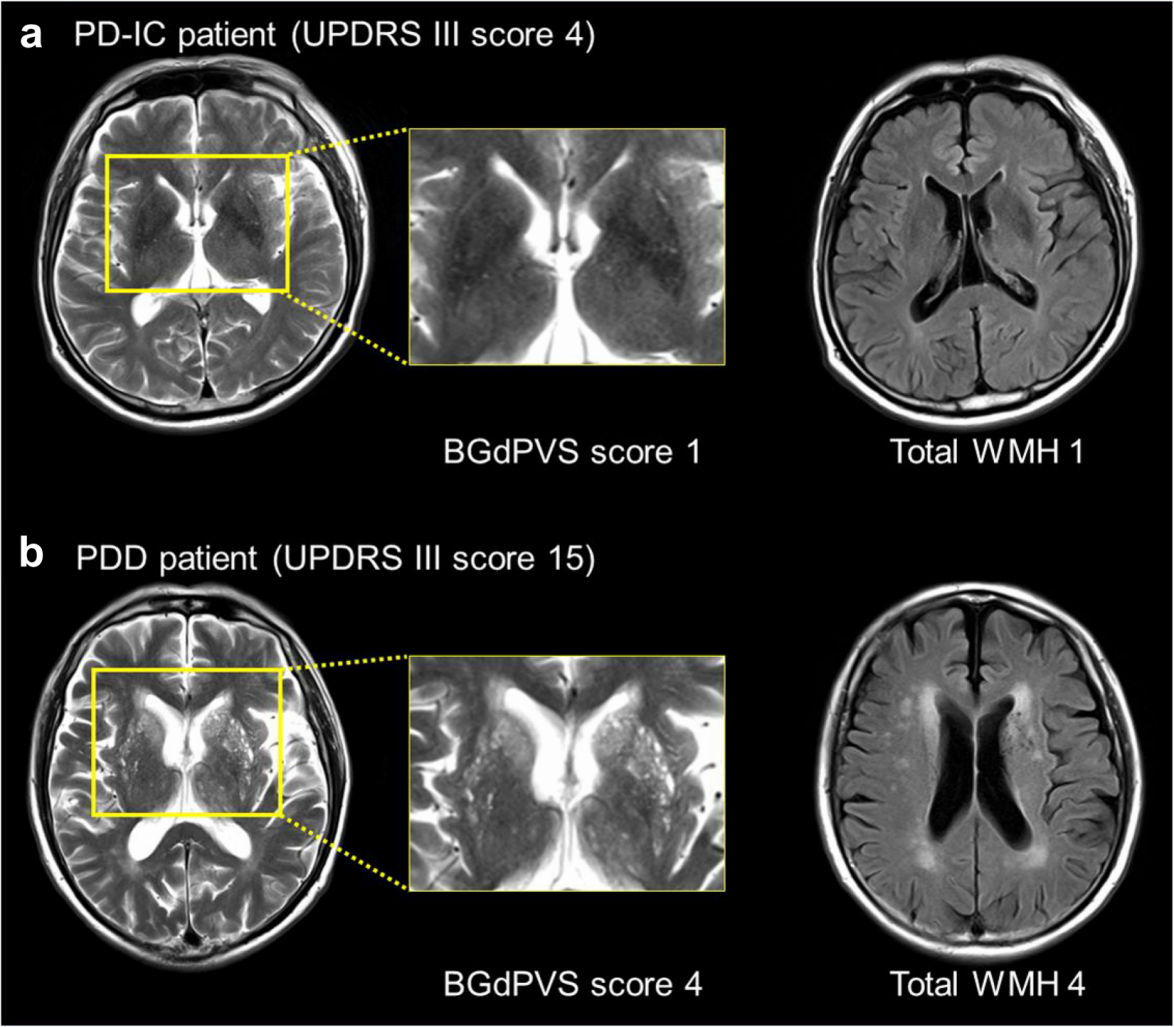
Representative cases showing the associations of BGdPVS and WMH with cognitive status and motor symptoms. **a** A 72-year-old male diagnosed as PD-IC, with a UPDRS III score of 4. The patient did not have a history of hypertension or SH. The axial T2-weighted images show a BGdPVS score of 1. On FLAIR images, the total Fazekas score of WMH was 1. **b** A 79-year-old male diagnosed with PDD, with a UPDRS III score of 15. The patient had hypertension and SH. The axial T2-weighted images show BGdPVS score of 4. On FLAIR images, the total Fazekas score of WMH was 4. Abbreviations: BG basal ganglia, dPVS dilated perivascular spaces, FLAIR fluid-attenuated inversion recovery, PD-IC PD with intact cognition, PDD PD with dementia, SH supine hypertension, UPDRS III Unified Parkinson’s Disease Rating Scale Part III, WMH white-matter hyperintensities.

Representative cases of BGdPVS and WMH being associated with cognition and motor symptoms are shown in Fig. 2.

#### Reanalysis excluding PDD patients and combining them with PD-MCI patients

The path analysis showed nearly identical trends for the original analysis in which PD-MCI and PDD patients were analyzed separately and the reanalysis in which PDD patients were either excluded or combined with PD-MCI under a single label (Supplementary Tables 4 and 5, respectively).

## Discussion

The present study examined the clinical effect of dPVS related to vascular and autonomic risk factors, RBD, APOE ε4 genotype, and WMH, with adjustment of known demographic and clinical risk factors. Our study resulted in three major findings. First, among the various clinical risk factors, hypertension and SH were significantly associated with BGdPVS. Second, BGdPVS mediated the effect of hypertension and SH on cognitive status via WMH or directly on motor symptoms without mediation of WMH. Third, the effect of WMH on cognition was driven by periventricular WMH, while deep WMH did not significantly affect cognition.

In line with previous results^2,9^, hypertension was significantly associated with higher BGdPVS and WMH in our study. More importantly, we found SH to be a novel risk factor for BGdPVS and an indirect one for WMH. Over the past few decades, OH rather than SH has received more attention as a risk factor for WMH, which is itself an imaging marker for small-vessel disease^12,19,20^. However, most studies did not consider the coexistence of SH^19,20^, while others suggested a greater association between SH and WMH than between OH and WMH^12,21^. Our study supports the latter studies, as our findings suggest SH to be more important in the pathogenesis of small-vessel disease. The vascular damage caused by high blood pressure when OH patients have SH may be more crucial in the development of cerebral small-vessel disease than the ischemic damage due to episodic hypotension associated with OH^22^. A recent study also found that SH in patients with OH was associated with a higher burden of target organ damage including cerebral small-vessel disease^23^.

In our study, WMH was significantly associated with only cognitive status, while BGdPVS was associated with both cognitive and motor symptoms. The association between WMH and cognition has been relatively consistent in previous literature^24,25^, while reports on its association with motor symptoms have been conflicting^26,27^. Therefore, BGdPVS may serve as a more useful imaging marker when predicting both symptoms. The effect of BGdPVS was mediated via WMH for cognition, whereas it directly affected motor function without WMH mediation. Although the reason for this phenomenon is unclear, a previous autopsy study of patients with parkinsonian symptoms reported decreased neuronal and axonal densities with reactive gliosis in BG adjacent to BGdPVS^28^. As BG plays a crucial role in motor function, we can postulate that parenchymal alterations accompanying BGdPVS might directly worsen motor symptoms in PD.

Our results were also similar to previous findings that suggested periventricular WMH rather than deep WMH as being associated with cognition in various populations^24,25^. Periventricular WM contains a higher density of long-association and projection fiber tracts that connect with more distant cortical areas and subcortical regions than the more superficially located deep WM as its fibers radiate to the cortical areas with a fan-like shape^29^. Given that effective communications with long distant cortical areas and subcortical regions are crucial to maintaining a normal cognitive process^30^, an association between periventricular WMH and cognition is understandable. Another possible explanation is that periventricular WMH may disrupt periventricular corticopetal cholinergic nerve fibers arising from the nucleus basalis of Meynert, which plays an important role in cognition^31^.

In contrast to previous results^32,33^, we failed to find significant associations between the presence of RBD with poor cognitive and motor symptoms. We also could not associate RBD and dPVS burden, while some previous studies have reported decreased sleep efficiency in PD patients with RBD^34^ and poor sleep efficiency with increased dPVS burden^13^, possibly due to decreased glymphatic function that increases during sleep^35^. These discordant results might be, at least in part, attributable to the inclusion of various clinical and imaging variables as covariates in our study, because when only age, sex, and education were included as covariates, RBD was found as an independent predictor of PD-MCI (*β* = 0.440, *P* = 0.044, not shown). However, as the association of RBD with WMH or dPVS has seldom been studied, further studies are warranted.

The presence of the APOE *ε*4 allele was also not associated with cognitive and motor symptoms nor imaging markers in our study. Although the APOE *ε*4 allele is thought to play an important role in the cognitive impairment of PD^8^, some studies have failed to find a significant contribution of the APOE *ε*4 allele in the cognitive decline of PD^36^. Reports about the APOE ε4 allele and WMdPVS are also inconsistent: significant association was reported in an autopsy study^37^, while an MRI study failed to find any such associations^38^. Inherent limits due to the spatial resolution of MRI and visual assessment of dPVS with a 5-point scale might account for these inconsistent results. Objective and quantitative assessment of dPVS imaged with high-resolution MRI might provide more conclusive results.

Although WMdPVS and deep WMH burdens did not significantly affect cognitive or motor symptoms in our PD patients, it is noteworthy that they show the same risk factors as well as different risk factors with BGdPVS and periventricular WMH, respectively. These findings suggest a common and distinct pathophysiology of dPVS and WMH according to location, and further studies are required to elucidate this possibility.

There are several limitations to our study. First, it is a cross-sectional, retrospective study, with a small number of PDD patients. Therefore, prospective studies with larger populations are warranted. Second, we only performed a qualitative analysis of dPVS and WMH. Although our analyzing methods are simple to perform with high interrater reliability, which allows their easy application in practical clinical workflow, future studies with objective and quantitative assessments of dPVS may offer new insights into the pathophysiology and clinical implications of dPVS. Therefore, automated and easy-to-use methods should continue to be developed. Third, because path analysis cannot be used to establish causality between variables^39^, future longitudinal studies are warranted to assess the causality between dPVS, WMH, and cognitive or motor symptoms in PD. Fourth, because dPVS has been shown to increase with age^40^, the older age of PD patients with cognitive impairment may be a confounding factor. Although adjustments were made for age in the analysis, future studies still need to consider the association between dPVS and age. Fifth, we used rather liberal criteria for defining “abnormal” outcomes of each neuropsychological test by using the one-standard-deviation cut-off value as well as for diagnosing “PD–MCI” with the abbreviated assessment of level I criteria, although these criteria were considered acceptable by the Movement Disorder Society Task Force^41^. Therefore, our results must be interpreted with caution as false-positive diagnoses cannot be ruled out for PD-MCI. Sixth, because MRI exams were usually performed to diagnose PD and neuropsychological tests were usually performed when patients had cognitive complaints, most patients who underwent both MRI and neuropsychological tests were PD-MCI patients and the proportions of PD-IC and PDD patients in our dataset were relatively small, which may have led to biased results. Despite these limitations, the strength of our study is that we found significant associations between hypertension and SH with BGdPVS, WMH, and cognitive or motor symptoms in PD patients even after adjusting for known clinical and imaging risk factors. Our results also have clinical implications as hypertension and SH are suggested as possible therapeutic targets to prevent or slow down the deterioration of cognitive and motor symptoms in PD patients, although a longitudinal study that assesses the effect of these treatments is warranted.

In conclusion, BGdPVS mediates the effect of hypertension and SH on cognitive impairment via total and periventricular WMH, whereas it is directly associated with severe motor symptoms. Our results indicate that BGdPVS adversely affects both cognitive and motor symptoms in PD and suggest SH as a novel risk factor for BGdPVS.

## Methods

### Study population

This study was approved by the Institutional Review Board of the Catholic University of Korea, Catholic Medical Center, Seoul St. Mary’s Hospital, and was issued a waiver of informed consent due to its retrospective design. PD patients who underwent MRI from April 2015 to August 2018 and who were assessed for cognitive status and motor symptoms within six months of MRI were selected from a prospectively collected movement disorders and dementia registry of a single tertiary hospital. Exclusion criteria were as follows: (1) incomplete clinical data (*n* = 33) and (2) the presence of large old infarcts or hemorrhage on MRI (*n* = 5). Thus, a total of 154 patients (79 males; mean age ± standard deviation, 70.2 ± 9.0) were included in the final study population (Supplementary Fig. 2). As MRI exams are usually performed when diagnosing PD in our institution, most of the patients enrolled in our study were relatively newly diagnosed PD patients.

PD was diagnosed according to the United Kingdom PD Society Brain Bank. Motor symptoms were assessed using the Unified Parkinson’s Disease Rating Scale Part III (higher scores represent more severe motor symptoms). In patients taking medication (*n* = 38), assessments were performed in the medication-on state. Cognitive status was determined using the Seoul Neuropsychological Screening Battery^42^, and the outcomes were defined as abnormal when the scores of each test were below the one-standard-deviation of the age-, sex-, and education-matched norms. PD-mild cognitive impairment (PD-MCI) and PD dementia (PDD) were diagnosed according to the Movement Disorder Society Task Force diagnostic criteria for PD-MCI level I and probable PDD, respectively^41,43^. PD medication doses were calculated as levodopa equivalents. The presence of vascular risk factors and APOE *ε*4 allele was assessed. Depressive symptoms were evaluated using the Geriatric Depression Scale or its short form with a cut-off value of 9/10 or 4/5 for defining depression, respectively^44^. The REM Sleep Behavior Disorder Screening Questionnaire (RBDSQ) with the primary question on RBD (question 1) of the Mayo Sleep Questionnaire (MSQ) or polysomnography was used to determine the presence of RBD. Patients with RBDSQ scores of 5 or more with affirmative responses to the MSQ question 1^45^ or polysomnographic REM sleep without atonia^46^ were defined as having RBD. Olfactory function was also assessed using the Cross-Cultural Smell Identification Test (higher scores represent higher performances). More details on the diagnostic criteria for vascular risk factors and cognitive status are in Supplementary Methods.

To diagnose OH and SH, the head-up tilt test was performed. Systolic and diastolic blood pressures were measured after patients had been in the supine position for 20 min and the lowest systolic and diastolic blood pressures were selected among measurements made 3 or 5 min after patients had begun the 60° tilted position, respectively. Then, orthostatic changes in the systolic (*Δ*SBP) and diastolic (*Δ*DBP) blood pressures were calculated. SH was defined with a SBP of 150 mm Hg or higher, or a DBP of 90 mm Hg or higher, with patients in the supine position^47^. OH was defined as an orthostatic ΔSBP ≥ 20 mmHg or ΔDBP ≥ 10 mmHg in patients without SH and as an orthostatic ΔSBP ≥ 30 mmHg or ΔDBP ≥ 15 mmHg in patients with SH^48^.

### MRI acquisition

MRI scans were acquired using a 3 T scanner (MAGNETOM Verio; Siemens Healthineers Sector, Erlangen, Germany) with a 12-channel coil. The MRI imaging protocol included two-dimensional axial T2-weighted and FLAIR images (parameters in Supplementary Methods).

### Analysis of dPVS

dPVS were assessed on T2-weighteed images according to STRIVE recommendations^49^, and rated in the basal ganglia (BG) and white matter (WM), respectively, using a validated 5-point visual rating scale (0 = absent dPVS, 1 = less than 10 dPVS, 2 = 11–20 dPVS, 3 = 21–40 dPVS, 4 = more than 40 dPVS)^50^ by a neuroradiologist with eight years of experience who was blinded to clinical data. The number of dPVS was counted in each hemisphere, and the highest score was recorded. Another neuroradiologist with 14 years of experience who was also blinded to clinical data rated the BGdPVS and WMdPVS in 30 randomly selected patients to assess interobserver agreements.

### Analysis of WMH

WMH were defined as hyperintense white-matter lesions on FLAIR images according to the STRIVE criteria^49^, and graded in accordance with the Fazekas scale by a neuroradiologist with eight years of experience who was blinded to clinical data. Periventricular and deep WMHs were rated separately. Periventricular WMH were scored as follows: 0 = absence, 1 = caps or pencil-thin lining, 2 = smooth halo, and 3 = irregular WMH extending into deep white matter. Deep WMH were scored as follows: 0 = absence, 1 = punctate, 2 = early confluent, and 3 = confluent. The scores for periventricular and deep WMH were added to calculate the total Fazekas score (0–6)^51^. ﻿

### Statistical analysis

According to normality test results, quantitative data were analyzed with either ANOVA or the Kruskal–Wallis test to compare the three groups. The Chi-squared test or Fisher’s exact test was used to analyze categorical data. A post hoc analysis was also performed using the Mann–Whitney *U* test, Chi-squared test, or Fisher’s exact test with Bonferroni-like adjustment for multiple comparisons. Inter-observer agreements for dPVS rating were analyzed using the weighted Cohen kappa coefficient.

Path analyses were performed to evaluate whether dPVS mediated the effects of vascular risk factors (hypertension, diabetes mellitus, and dyslipidemia), autonomic abnormalities (OH and SH), APOE ε4 status, and RBD on total WMH and cognition. Path analysis was used to simultaneously consider the direct, indirect, and total effects of predictors on outcomes through mediators. Cognitive status was labeled as the outcome (PD-intact cognition [PD-IC] was labeled as 0, PD-MCI as 1, and PDD as 2). Age, sex, education, disease duration, levodopa-equivalent dose, the Cross-Cultural Smell Identification Test score, and depression were used as covariates. An identical process was performed by using either periventricular WMH or deep WMH as a variable instead of total WMH, to assess the differential role of WMH on cognition status according to location.

To evaluate whether dPVS mediates the effects of clinical risk factors on motor symptoms, path analyses were performed identically for cognitive status, except when the Unified Parkinson’s Disease Rating Scale Part III score was considered the outcome.

In addition, because there were few PDD patients available for analysis, we needed to determine if this small group inappropriately drove the results. The data were reanalyzed, first excluding PDD, and second combining PD-MCI and PDD under a single label.

The variance-inflation factor was used to detect multicollinearity between variables. *A P* value <0.05 was considered statistically significant. All statistical analyses were performed using SPSS (version 24; IBM Corp., Armonk, NY, USA).

### Reporting summary

Further information on research design is available in the Nature Research Reporting Summary linked to this article.

## Supplementary information

Supplementary Information

Reporting Summary

## Data Availability

Our anonymized data can be obtained by any qualified investigators for the purposes of replicating procedures and results, after ethics clearance by the Catholic University of Korea and approval by all authors (contact to nyshin@catholic.ac.kr).
