# Supplementary material for: Adverse effects of hypertension, supine hypertension, and perivascular space on cognition and motor function in PD
Source: NPJ Parkinsons Dis. 2021 Aug 10;7:69. doi: 10.1038/s41531-021-00214-6 (PMC8355129; doi:10.1038/s41531-021-00214-6)
Supplement: Supplementary file 1 — Supplementary Information [file 41531_2021_214_MOESM1_ESM.pdf]

Supplementary Materials

Supplementary Methods

Diagnostic criteria for vascular risk factors and cognitive status

Definitions of vascular risk factors

Hypertension was determined with the use of antihypertensive treatment before admission or repeated measurements of a systolic pressure > 140mm Hg or diastolic pressure > 90mm Hg. Diabetes mellitus was determined with a fasting blood glucose level > 126 mg/dL or a history of diabetes treatment. Dyslipidemia was determined with a total cholesterol level > 200 mg/dL or a low-density lipoprotein cholesterol > 130 mg/dL at the time of presentation or use of lipid-lowering agents.

Definition of cognitive status

Information about memory problems or other deficits in daily lives was gathered through interviews with patients or their caregivers. Cognitive status was diagnosed in consensus between two neurologists and one neuropsychologist based on a neuropsychological battery and physician-administered neurologic examination.
The Seoul Neuropsychological Screening Battery was used to assess cognitive status. [1] The Seoul Neuropsychological Screening Battery includes cognitive subsets for attention and working memory (forward and backward digit span, Korean version of the Tail Making Test for the Elderly [K-TMT-E] Part A, and Korean version of the Color Word Stroop test), executive function (phonemic and sematic Controlled Oral Word Association Test, and K-TMT-E Part B), language (Korean version of the Boston Naming Test [K-BNT]), memory (Seoul Verbal Learning Test; immediate recall, 20-minute delayed recall, and recognition for verbal memory, and RCFT; immediate recall, 20-minute delayed recall, and recognition for visual memory), and visuospatial function (Rey Complex Figure Test [RCFT] copy). General cognitive abilities were assessed using the Korean version of the Mini-Mental State Examination (K-MMSE). Outcomes were defined as abnormal when the scores of each test were 1 standard deviation below the age-, sex-, and education-matched norms. PD-associated mild cognitive impairment (PD-MCI) and PD dementia (PDD) were diagnosed according to the Movement Disorder Society Task Force diagnostic criteria for PD-MCI level I and probable PDD, respectively. [2, 3] For a diagnosis of PD-MCI by level I criteria, impairment had to be observed for global cognitive function (K-MMSE scores of <27) or on at least two tests within one domain or across different domains without significant impairment on daily life caused by cognitive deficits. For a diagnosis of probable PDD, impairment had to be observed on at least two of four core cognitive domains (i.e., attention and working memory,

executive function, memory, and visuospatial function) which impaired daily life. Patients with Clinical Dementia Rating (CDR) scores of ≥ 1 or Korean-Instrumental Activities of Daily Living (K-IADL) scores of ≥ 0.35 were considered to have impaired daily lives. [4] The subtests from the same test were counted as one test. Therefore, a total of 9 tests were used to classify cognitive status.

MRI parameters

MRI scans were acquired using a 3T scanner (MAGNETOM Verio; Siemens Healthineers Sector, Erlangen, Germany) with a 12-channel coil. The two-dimensional T2-weighted turbo spin echo images were obtained with the following parameters: repetition time [TR]/echo time [TE] 4950/93 ms; field of view [FOV] 210 mm x 210 mm; matrix, 448 x 358; flip angle, 150°; slice thickness, 5 mm; gap, 1 mm; echo train length, 17; and number of excitations [NEX], 1. The fluid-attenuated inversion recovery (FLAIR) images were obtained with the following parameters: TR/TE 9000/99 ms; FOV 210 x 210 mm; matrix, 320 x 224; flip angle, 150°; slice thickness, 5 mm; gap, 1 mm; echo train length, 13; NEX, 1.

Supplementary References

1. Kang Y, Jahng S, Na D. Seoul Neuropsychological Screening Battery. (SNSB-II): Professional Manual. Incheon: Human Brain Research and Consulting 2012.
2. Emre M, Aarsland D, Brown R, et al. Clinical diagnostic criteria for dementia associated with Parkinson's disease. Mov Disord 2007;22(12):1689-1707; quiz 1837.
3. Litvan I, Goldman JG, Troster AI, et al. Diagnostic criteria for mild cognitive impairment in Parkinson's disease: Movement Disorder Society Task Force guidelines. Mov Disord 2012;27(3):349-356.
4. Chin J, Park J, Yang SJ, et al. Re-standardization of the Korean-Instrumental Activities of Daily Living (K-IADL): Clinical Usefulness for Various Neurodegenerative Diseases. Dement Neurocogn Disord 2018;17(1):11-22.

Supplementary Figure 1. Schematic diagram of path analyses for cognitive status and motor symptoms when periventricular WMH were used as a variable.

Vascular risk factors (hypertension, diabetes mellitus, dyslipidemia), autonomic risk factors (SH, OH), APOE ε4 status, and RBD were entered as predictors. BGdPVS, WMdPVS, and periventricular WMH were entered as mediators. Age, sex, education, disease duration, levodopa-equivalent dosage, CCSIT score, and depression were entered as covariates. Numbers on the paths are standardized.

Abbreviations: BG = basal ganglia, dPVS = dilated perivascular space, OH = orthostatic hypotension, RBD = rapid eye movement sleep behavior disorder, SH = supine hypertension, WMH = white matter hyperintensities.

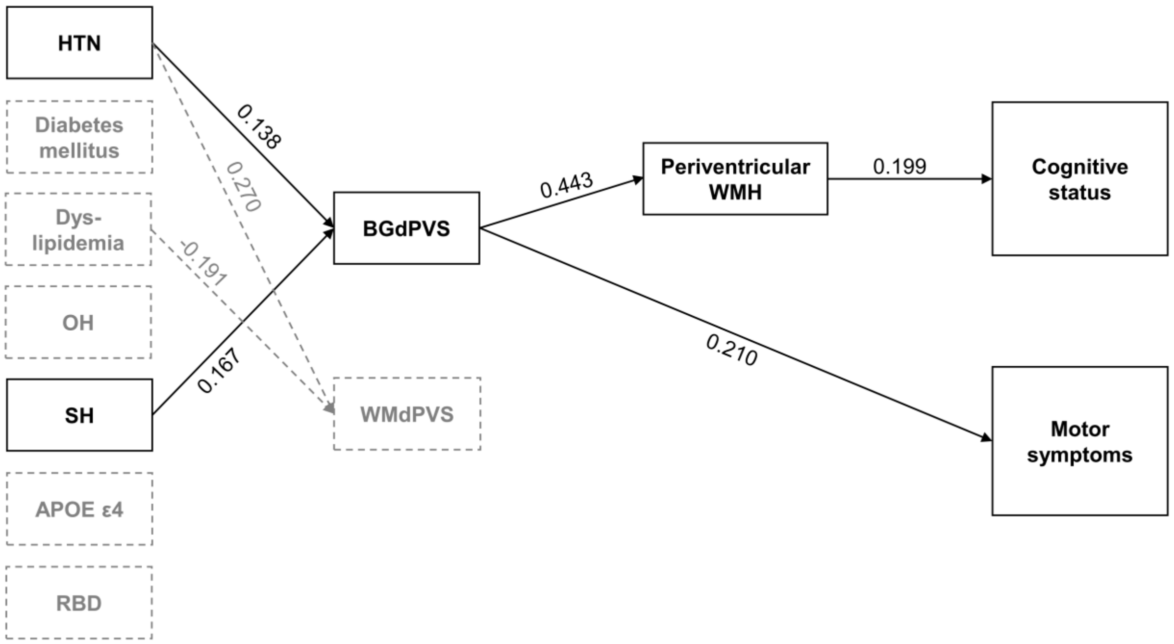

Supplementary Figure 2. Patient flowchart.
Abbreviations: PDD, PD with dementia; PD-IC, PD with intact cognition; PD-MCI, PD with mild cognitive impairment.

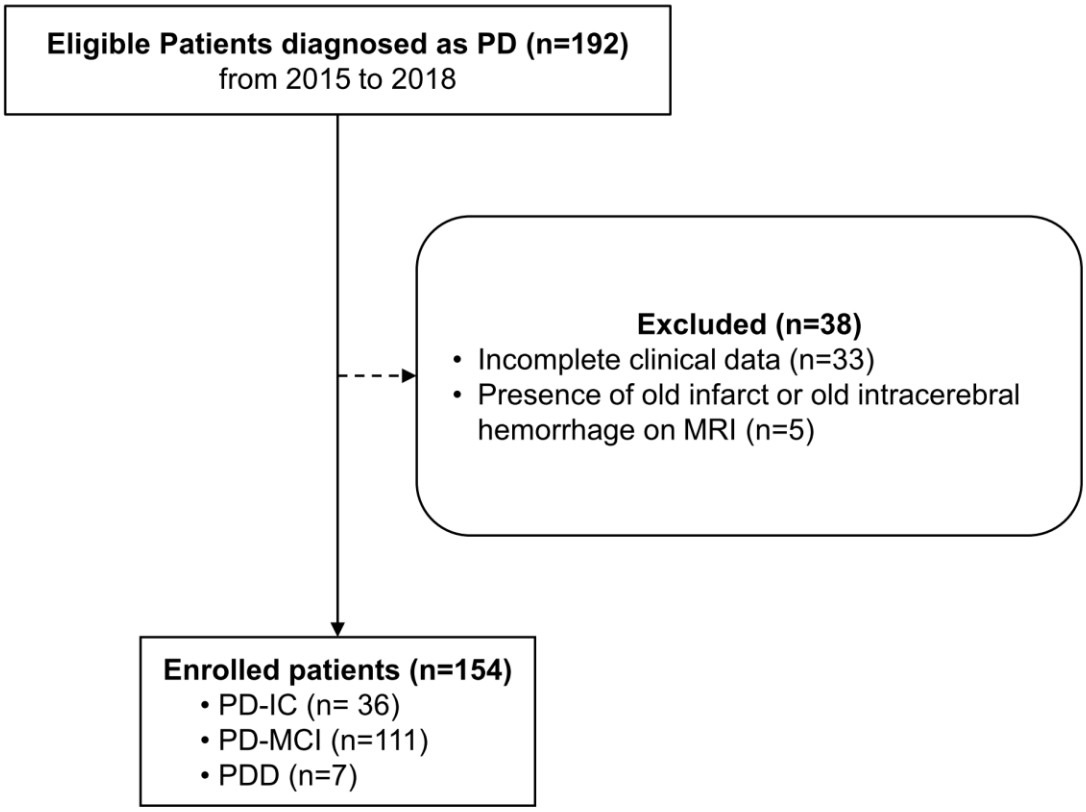

Supplementary Table 1. BGdPVS, WMdPVS, total WMH, periventricular WMH, and deep WMH scores according to cognitive status.

	PD-IC (n = 36)	PD-MCI (n = 111)	PDD (n = 7)
BGdPVS			
0 (absent)	1 (2.8)	0 (0)	0 (0)
1 (less than 10)	20 (55.6)	58 (52.3)	0 (0)
2 (11 to 20)	9 (25.0)	26 (23.4)	3 (42.8)
3 (21 to 40)	4 (11.1)	13 (11.7)	2 (28.6)
4 (more than 40)	2 (5.6)	14 (12.6)	2 (28.6)
WMdPVS			
0 (absent)	0 (0)	0 (0)	0 (0)
1 (less than 10)	4 (11.1)	15 (13.5)	0 (0)
2 (11 to 20)	13 (36.1)	28 (25.3)	3 (42.8)
3 (21 to 40)	9 (25.0)	30 (27.0)	2 (28.6)
4 (more than 40)	10 (27.8)	38 (34.2)	2 (28.6)
Total WMH			
0	1 (2.8)	2 (1.8)	0 (0)
1	12 (33.4)	20 (18.0)	1 (14.3)
2	16 (44.4)	47 (42.3)	2 (28.6)
3	4 (11.1)	25 (22.5)	0 (0)
4	3 (8.3)	13 (11.8)	4 (57.1)
5	0 (0)	4 (3.6)	0 (0)
6	0 (0)	0 (0)	0 (0)
Periventricular WMH			
0 (absence)	1 (2.8)	3 (2.7)	0 (0)
1 (punctate)	27 (75.0)	63 (56.8)	3 (42.9)
2 (early confluent)	7 (19.4)	36 (32.4)	0 (0)
3 (confluent)	1 (2.8)	9 (8.1)	4 (57.1)
Deep WMH			
0 (absence)	14 (38.8)	24 (21.6)	1 (14.3)
1 (punctate)	20 (55.6)	75 (67.6)	6 (85.7)
2 (early confluent)	2 (5.6)	11 (9.8)	0 (0)
3 (confluent)	0 (0)	1 (0.9)	0 (0)

Data are expressed as numbers with percentages in parentheses.

Abbreviations: BG, basal ganglia; dPVS, dilated perivascular space; PDD, PD with dementia; PD-IC, PD with intact cognition; PD-MCI, PD with mild cognitive impairment; WM, white matter; WMH, white matter hyperintensities.

Supplementary Table 2. Effects of predictors on cognitive status and motor symptoms through mediators when periventricular WMH was used as a variable.

	BGdPVS			WMdPVS			Periventricular WMH			Cognitive status			UPDRSIII		
	β	SE	*P*	*β*	SE	*P*	*β*	SE	*P*	*β*	SE	*P*	*β*	SE	*P*
Hypertension	0.138	0.148	0.045	0.270	0.158	< 0.001	0.091	0.093	0.165	-0.038	0.083	0.648	-0.131	1.443	0.086
Diabetes mellitus	-0.071	0.194	0.313	-0.140	0.206	0.069	-0.029	0.117	0.651	-0.019	0.104	0.817	0.014	1.809	0.855
Dyslipidemia	-0.070	0.148	0.318	-0.191	0.157	0.013	-0.017	0.090	0.791	-0.060	0.080	0.458	-0.100	1.391	0.180
OH	-0.097	0.190	0.170	-0.025	0.202	0.744	0.077	0.113	0.231	-0.009	0.102	0.910	0.120	1.764	0.108
SH	0.167	0.266	0.016	0.083	0.283	0.276	0.047	0.161	0.462	0.066	0.145	0.415	-0.050	2.504	0.499
RBD	0.047	0.160	0.514	0.079	0.170	0.316	-0.028	0.095	0.671	0.117	0.085	0.154	0.033	1.476	0.664
APOE ε4 carrier	0.103	0.183	0.136	-0.052	0.195	0.491	0.012	0.110	0.855	-0.023	0.098	0.766	-0.089	1.698	0.222
BGdPVS							0.443	0.048	<0.001	0.038	0.047	0.711	0.210	0.822	0.025
WMdPVS							-0.070	0.045	0.294	-0.012	0.040	0.889	-0.101	0.697	0.187
Periventricular WMH										0.199	0.072	0.048	0.038	1.245	0.685

Age, sex, years of education, disease duration, levodopa-equivalent dose, CCSIT score, and the presence of depression were used as covariates.

Abbreviations: *β*, standardized beta coefficient; BG, basal ganglia; dPVS, dilated perivascular spaces; OH, orthostatic hypotension; RBD, REM sleep behavior disorder; SE, standard error; SH, supine hypertension; UPDRS III, Unified Parkinson's Disease Rating Scale III; WM, white matter; WMH, white matter hyperintensities.

Supplementary Table 3. Effects of predictors on cognitive status and motor symptoms through mediators when deep WMH was used as a variable.

	BGdPVS			WMdPVS			Deep WMH			Cognitive status			UPDRSIII		
	β	SE	P	β	SE	P	β	SE	P	β	SE	P	β	SE	P
Hypertension	0.138	0.148	0.045	0.270	0.158	< 0.001	-0.034	0.094	0.675	-0.016	0.083	0.850	-0.131	1.428	0.083
Diabetes mellitus	-0.071	0.194	0.313	-0.140	0.206	0.069	0.080	0.119	0.308	-0.034	0.105	0.676	0.020	1.805	0.785
Dyslipidemia	-0.070	0.148	0.318	-0.191	0.157	0.013	0.048	0.091	0.540	-0.069	0.081	0.396	-0.096	1.386	0.196
OH	-0.097	0.190	0.170	-0.025	0.202	0.744	-0.218	0.115	0.005	0.032	0.105	0.699	0.102	1.791	0.179
SH	0.167	0.266	0.016	0.083	0.283	0.276	0.014	0.164	0.856	0.074	0.145	0.364	-0.047	2.488	0.524
RBD	0.047	0.160	0.514	0.079	0.170	0.316	0.007	0.097	0.927	0.110	0.086	0.181	0.032	1.468	0.665
APOE ε4 carrier	0.103	0.183	0.136	-0.052	0.195	0.491	-0.010	0.112	0.892	-0.020	0.099	0.802	-0.090	1.690	0.217
BGdPVS							0.293	0.048	0.001	0.091	0.044	0.336	0.255	0.760	0.003
WMdPVS							0.023	0.046	0.775	-0.028	0.040	0.735	-0.102	0.692	0.182
Deep WMH										0.119	0.071	0.152	-0.096	1.217	0.204

Age, sex, years of education, disease duration, levodopa-equivalent dose, CCSIT score, and the presence of depression were used as covariates.

Abbreviations: *β*, standardized beta coefficient; BG, basal ganglia; dPVS, dilated perivascular spaces; OH, orthostatic hypotension; RBD, REM sleep behavior disorder; SE, standard error; SH, supine hypertension; UPDRS III, Unified Parkinson's Disease Rating Scale III; WM, white matter; WMH, white matter hyperintensities.

Supplementary Table 4. Effects of predictors on cognitive status and motor symptoms through mediators when total WMH was used as a variable after excluding PDD patients.

	BGdPVS			WMdPVS			Total WMH			Cognitive status			UPDRSIII		
	β	SE	*P*	*β*	SE	*P*	*β*	SE	*P*	*β*	SE	*P*	*β*	SE	*P*
Hypertension	0.146	0.077	0.042	0.146	0.083	0.042	0.047	0.077	0.542	-0.044	0.088	0.618	-0.095	0.774	0.248
Diabetes mellitus	-0.089	0.078	0.257	-0.089	0.085	0.295	-0.003	0.077	0.970	-0.115	0.087	0.189	0.012	0.772	0.879
Dyslipidemia	-0.032	0.079	0.682	-0.175	0.085	0.037	0.056	0.077	0.474	-0.071	0.088	0.425	-0.086	0.782	0.300
OH	-0.048	0.076	0.524	-0.060	0.082	0.466	-0.015	0.074	0.835	0.076	0.084	0.368	-0.140	0.742	0.080
SH	0.172	0.076	0.017	0.026	0.082	0.747	0.027	0.074	0.713	0.044	0.084	0.610	-0.019	0.739	0.812
RBD	0.040	0.079	0.615	-0.010	0.085	0.906	-0.021	0.076	0.788	0.158	0.087	0.072	0.079	0.759	0.329
APOE ε4 carrier	0.108	0.077	0.161	-0.072	0.083	0.388	0.018	0.075	0.807	0.040	0.086	0.641	-0.117	0.761	0.148
BGdPVS							0.423	0.087	<0.001	-0.064	0.107	0.549	0.232	0.952	0.023
WMdPVS							-0.035	0.080	0.668	0.023	0.091	0.803	-0.124	0.808	0.150
Total WMH										0.203	0.100	0.031	-0.075	0.883	0.426

Age, sex, years of education, disease duration, levodopa-equivalent dose, CCSIT score, and the presence of depression were used as covariates.

Abbreviations: *β*, standardized beta coefficient; BG, basal ganglia; dPVS, dilated perivascular spaces; OH, orthostatic hypotension; RBD, REM sleep behavior disorder; SE, standard error; SH, supine hypertension; UPDRS III, Unified Parkinson's Disease Rating Scale III; WM, white matter; WMH, white matter hyperintensities.

Supplementary Table 5. Effects of predictors on cognitive status and motor symptoms through mediators when total WMH was used as a variable after PD-MCI and PDD were combined under a single label.

	BGdPVS			WMdPVS			Total WMH			Cognitive status			UPDRSIII		
	β	SE	*P*	*β*	SE	*P*	*β*	SE	*P*	*β*	SE	*P*	*β*	SE	*P*
Hypertension	0.138	0.074	0.045	0.138	0.081	0.045	0.037	0.074	0.620	-0.048	0.086	0.582	-0.109	0.746	0.174
Diabetes mellitus	-0.107	0.075	0.159	-0.123	0.082	0.134	-0.002	0.073	0.983	-0.089	0.085	0.298	0.029	0.737	0.711
Dyslipidemia	-0.032	0.076	0.670	-0.162	0.082	0.051	0.025	0.074	0.735	-0.0.9	0.086	0.360	-0.077	0.744	0.333
OH	-0.041	0.074	0.580	-0.053	0.080	0.508	-0.025	0.071	0.721	0.066	0.082	0.428	-0.135	0.713	0.079
SH	0.167	0.075	0.016	0.055	0.082	0.503	0.008	0.073	0.918	0.045	0.084	0.595	-0.004	0.729	0.954
RBD	0.017	0.076	0.825	0.000	0.082	0.997	-0.056	0.073	0.445	0.145	0.085	0.090	0.057	0.732	0.465
APOE ε4 carrier	0.081	0.074	0.274	-0.082	0.080	0.308	-0.015	0.072	0.840	0.026	0.084	0.752	-0.111	0.722	0.153
BGdPVS							0.440	0.084	<0.001	-0.039	0.107	0.719	0.240	0.928	0.017
WMdPVS							-0.036	0.078	0.641	0.018	0.090	0.842	-0.119	0.779	0.156
Total WMH										0.206	0.099	0.027	-0.068	0.857	0.458

Age, sex, years of education, disease duration, levodopa-equivalent dose, CCSIT score, and the presence of depression were used as covariates.

Abbreviations: *β*, standardized beta coefficient; BG, basal ganglia; dPVS, dilated perivascular spaces; OH, orthostatic hypotension; RBD, REM sleep behavior disorder; SE, standard error; SH, supine hypertension; UPDRS III, Unified Parkinson's Disease Rating Scale III; WM, white matter; WMH, white matter hyperintensities.
